# Significant gender-specific difference in brain lateralization of schizophrenia patients assessed by new combined foot dominance scale

**DOI:** 10.3389/fpsyt.2023.1276920

**Published:** 2023-11-30

**Authors:** Katerina Akabalieva

**Affiliations:** Department of Apasychiatry and Amaedical Apasychology, Faculty of Medicine, Medical University Sofia, Sofia, Bulgaria

**Keywords:** schizophrenia, dysontogenesis, laterality, foot dominance, biological marker, gender dimorphism

## Abstract

**Objective:**

Reduced hemispheric asymmetry has been identified as a potential risk factor for schizophrenia, characterized by diminished brain lateralization and a lack of dominance in the left hemisphere. Moreover, there is growing evidence of disrupted connectivity between various cortical regions. This study aimed to investigate gender differences in left-footedness as a potential biological marker for neuronal dysontogenesis in individuals with schizophrenia and control subjects.

**Materials and methods:**

A New Combined Foot Dominance Scale (14 foot tests), comprising a Modified Chapman & Chapman Subscale (10 foot tests) and a Complex Tasks Subscale (four foot tests) was administered as performance tasks in 180 subjects [98 schizophrenia patients with mean age 34.45 years (SD = 15.67, range 23–79) for men and 42.20 years (SD = 11.38, range 21–63) for women and 82 controls with a mean age 34.70 years (SD = 16,82, range 18–79) for men and 44.50 years (SD = 10.73, range 23–67)]. As our data are not continuous and lacks normal distribution, the non-parametric Mann–Whitney test was used for comparing categorical data.

**Results:**

The mean left-footedness, as assessed by the New Combined Foot Dominance Scale, is significantly higher in individuals with schizophrenia compared to control subjects. Our findings from inter-gender comparisons reveal that female schizophrenia patients exhibit a significantly greater average left-footedness than female control subjects, while in males no such a statistical significant difference is detected.

**Conclusion:**

Left foot dominance is higher in patients with schizophrenia than in control subjects and women contribute significantly more to this difference.

## Introduction

The concept of hemispheric lateralization began to play an increasingly important role in the neuropsychological and pathophysiological models of schizophrenia (1). The fact that lateralization is a fundamental characteristic and principle in brain functioning shows that disturbed brain asymmetry is somehow involved in defective neurontogenesis. Here, early life stress could be a factor disrupting typical brain development ultimately causing altered functional as well as structural hemispheric asymmetries. The study of functional lateralization (manual, foot, and eye dominance) amplifies the neuroontogenetic model of schizophrenia, adding explanations for early disorders of brain maturation.

In recent years, the hypothesis that schizophrenia is linked to abnormal cerebral lateralization has gained support by studies demonstrating anomalies in hand, eye, and foot preferences. Brain asymmetry has been observed at the structural level in the fetal brain of both humans and nonhuman primates (2) and appears to be disrupted in patients with autism spectrum disorder (ASD), schizophrenia, and developmental dyslexia (3). Reduction of brain asymmetry, primarily due to the failure of left-hemisphere dominance, has been documented in schizophrenia (4).

Footedness refers to the dominant or preferred foot in a context that necessitates choosing one of the feet for performing manipulative or mobilizing actions. Footedness is less influenced by cultural norms or social teaching compared to handedness for which influences of these factors have been documented repeatedly (5–8).

A recent meta-analysis confirms previous studies, which indicated that non-right-footedness prevalence was higher in males than in females in general population (9, 10). Another study found that men were more likely to be left-footed (11) and left- and mixed-footed than women (12) and that women were more likely to be left-eyed than men (13). Some studies conducted in different geographical regions of the world (14–16), found no gender difference in footedness and eyedness among the general population. A study on hand, foot, and eye dominance showed more left dominance in schizophrenia patients (17). A later study confirmed the excessive left or mixed preference for all three aspects of dominance—eye, foot, and hand—in schizophrenia patients in comparison to healthy controls (18, 19). Another study, however, could not confirm such a preference in patients with schizophrenia—neither for eye nor for foot dominance (20). Some authors found no significant excess of non-right foot dominance in schizophrenia patients vs. controls (21–23).

As far as we know there are few studies investigating gender differences in foot dominance in schizophrenia patients. In a prospective study of children, who develop schizophrenia spectrum disorders and those who did not develop, was found that males and females did not significantly differ in rates of left or mixed-handedness, footedness, and eye dominance.

This study is part of a larger investigation project on the intriguing relations between six groups of markers of neuronal dysontogenesis—left-handedness, left-footedness, left-eyedness, minor physical anomalies, digit ratio, and cognitive (attention and memory) deficit. The aim of the current article is to investigate gender differences in foot dominance as biological markers of neuronal dysontogenesis in schizophrenia patients and control subjects.

## Materials and methods

### Subjects

The study was conducted in the Clinic of Psychiatry at the University Hospital in Sofia and at the State Psychiatric Hospital in Radnevo. The sample included 98 (56 men, 42 women) consecutively admitted inpatients with schizophrenia with mean age 34.45 years (SD = 15.67, range 23–79) for men and 42.20 years (SD = 11.38, range 21–63) for women.

The patients satisfied the Diagnostic and Statistical Manual of Mental Disorders (DSM-V) criteria for a diagnosis of schizophrenia on the basis of case records review, DSM-V based semi-structured interview and information obtained from relatives for stronger validation of the diagnosis. In order to enhance the homogeneity of the schizophrenia group, potential subjects were excluded if they had a history of drug or alcohol abuse, identifiable neurological disorder (seizure disorder, head injury, multiple sclerosis, etc.), any signs of intellectual disability or somatic disorder with neurological components. Exclusion criteria were also any previous or present lower limb disorders hampering the performance of the foot tasks and any disease which affects balance or coordination.

The control group consisted of 82 subjects (30 men, 52 women) with a mean age 34.70 years (SD = 16,82, range 18–79) for men and 44.50 years (SD = 10.73, range 23–67) for women. Normality was defined as the absence of a previous or present psychiatric disorder. Controls satisfied exclusion criteria similar to those applied to the schizophrenia patients. In addition, for better separation between the control and the schizophrenia group, we implemented another exclusion criterion for controls—having a first-degree relative with a history of a psychotic disorder, major affective disorder, or suicide.

To avoid potential confound due to ethnic and racial differences related to lateralization both schizophrenia patients and controls were of Bulgarian origin. Individuals were excluded if their parental or grandparental ethnic group was other than Bulgarian.

The refusal rate of potential participants was insignificant [below 5%], excluding selectivity bias.

The study was approved by the Local Ethics Committee and all subjects gave written informed consent prior to participation.

### Instruments

#### New combined foot dominance scale

It includes two subscales:

##### Modified Chapman & Chapman subscale

Among the foot dominance instruments in the literature available to us, the most popular is the Chapman & Chapman questionnaire (5). It includes 11 foot performed activities: Kick as high as possible, Kick a ball, Arrange cubes, Going through cubes with a ball, Rolling a golf ball around a circle, Stamp out a cigarette, Balancing a rod on the thigh, Write your initials on the sand, Smooth the sand, Hopping on one foot, and Tapping out a rhythm.

Two modifications were made to the subscale:

- The task “Tapping out a rhythm of Yankee Doodle,” since this melody is unknown to Bulgarians, has been reformulated to “Tapping out the rhythm of a favorite song.”
- The task “Going through cubes with a ball” was excluded due to poor informativeness. The participants (mainly schizophrenic patients) could not complete the task by choosing only one foot to lead the ball and used both feet. This led to the false overuse of the category “Both equally,” which however did not reflect the actual dominant foot of the subject for this task.

##### Complex tasks subscale

It includes four foot dominance tests, measuring more complex tasks.

- Step up on a chair (assess spontaneity).
- Pick up object with toe (assess precision).
- Push shovel into the ground (assess strength).
- Standing on one foot (preferred foot for carrying the body weight; assess balancing).

Measuring these additional complex tasks allows for a subtle and comprehensive assessment of foot dominance.

The New Combined Foot Dominance Scale includes a total of 14 tasks, administered as performance assessments, not as preference questionnaires. Each test was performed twice and the subject was asked to perform the test again if there was any inconsistency in the preference.

Each foot test is rated: 0—Preference of right foot; 1—No preference (both feet equally); and 2—Preference of left foot. Each test score ranges from 0 to 2; the total score of the New Combined Foot Dominance Scale ranges from 0 to 28.

The refusal rate of potential participants was insignificant [below 5%], excluding selectivity bias.

All assessments were performed by the same examiner (KA).

### Statistical analysis

Data were analyzed with SPSS 25.0.

Parametric statistics (mean and standard deviation) were used for descriptive purposes.

As our data are not continuous and lack normal distribution, the non-parametric Mann–Whitney test for means difference between two independent groups was used.

The categories of the foot tests can be treated as ordinal data—graded, in ascending order of leftedness: 0—Preference of right foot; 1—No preference; and 2—Preference of left foot. Ordinal data allow calculating the mean left-footedness for every single foot test. A variable mean is usually more sensitive than its categories in detecting a difference between groups. Furthermore, it enables comparison of the important differences of the mean sum of left-footedness of the Modified Chapman & Chapman Subscale (10 foot tests), Complex Tasks Subscale (four foot tests), and the New Combined Foot Dominance Scale (14 foot tests) between schizophrenic and control subjects.

Statistical significance was defined as *p* < 0.05; two-tailed.

## Results

### Comparison of left-footedness between patients with schizophrenia and control subjects

The mean left-footedness of all the foot tests (except arrange cubes) of the *Combined Foot Dominance Scale* are higher in patients with schizophrenia vs. control subjects:

- The sum mean left-footedness of the *Chapman & Chapman subscale* is significantly higher in patients with schizophrenia vs. control subjects (4.10 vs. 2.51, *p* < 0.004, over 1.6 times increase).
- The sum mean left-footedness of the *Complex Task Subscale* is also significantly higher in patients with schizophrenia than in control subjects (2.64 vs. 1.98, *p* < 0.027, 1.3 times increase).

Importantly, the cumulative sum mean left-footedness of the *New Combined Foot Dominance Scale* is strongly significantly higher in patients with schizophrenia than in controls (6.77 vs. 4.47, *p* < 0.002, approximately 1.5 times increase).

This shows that the overall mean left-footedness is higher in patients with schizophrenia compared to control subjects.

However, when examining the mean left-footedness between patients with schizophrenia and control subjects, a notable difference emerges when considering gender. The comparison reveals significant distinctions between men and women.

### Comparison of mean left-footedness between patients with schizophrenia and control subjects by gender

Analyzing the breakdown by gender of the contribution (Table 1) to the pronounced disparity in mean left-footedness between patients with schizophrenia and control subjects reveals that women contribute significantly more to this difference.

**Table 1:** Comparison of *Mean Left-footedness* between patients with schizophrenia and control subjects by gender.

	Men (*N* = 86)						Women (*N* = 94)					
Foot tests	Schizophrenia (*n* = 56)		Controls (*n* = 30)		Statistical significance^*^		Schizophrenia (*n* = 42)		Controls (*n* = 52)		Statistical significance^*^	
	Mean	SD	Mean	SD	U	*p*	Mean	SD	Mean	SD	U	*p*
Chapman & Chapman subscale												
Kick as high as possible	0.41	0.78	0.40	0.81	822.0	0.821	0.52	0.89	0.25	0.65	947.5	0.107
Kick a ball	0.30	0.71	0.27	0.64	838.5	0.983	0.33	0.75	0.21	0.61	1032.5	0.450
Arrange cubes	0.31	0.67	0.43	0.77	755.5	0.486	0.38	0.76	0.38	0.77	1091.0	0.991
Rolling a golf ball around circle	0.27	0.68	0.27	0.69	817.0	0.903	0.48	0.86	0.15	0.54	916.0	**0.030**
Stamp out a cigarette	0.33	0.75	0.27	0.69	783.0	0.687	0.19	0.59	0.12	0.47	1051.0	0.493
Balancing a rod on the thigh	0.44	0.83	0.31	0.71	755.0	0.566	0.69	0.95	0.25	0.65	845.0	**0.011**
Write your initials on the sand	0.25	0.67	0.13	0.51	775.0	0.388	0.14	0.52	0.15	0.54	1086.0	0.920
Smooth the sand	0.44	0.83	0.23	0.63	749.0	0.303	0.33	0.75	0.25	0.65	1053.5	0.635
Hopping on one foot	0.75	0.97	0.50	0.78	737.5	0.342	0.86	1.00	0.37	0.77	825.0	**0.011**
Tapping out a rhythm	0.43	0.82	0.20	0.55	728.0	0.262	0.48	0.86	0.27	0.69	979.0	0.197
Subtotal mean left-footedness	3.85	5.11	2.69	4.87	650.0	0.288	4.40	5.09	2.40	4.60	723.0	**0.003**
Complex task subscale												
Step up on a chair	0.87	1.00	0.53	0.90	685.0	0.125	0.86	1.00	0.37	0.77	825.0	**0.011**
Pick up object with toe	0.42	0.80	0.33	0.71	768.0	0.721	0.43	0.83	0.23	0.61	996.0	0.263
Push shovel into the ground	0.68	0.96	1.03	1.00	686.5	0.103	0.71	0.97	0.42	0.82	933.0	0.119
Standing on one foot	0.57	0.79	0.47	0.73	757.0	0.564	0.93	0.95	0.73	0.91	971.0	0.305
Subtotal mean left-footedness	2.40	2.05	2.37	2.19	756.5	0.817	2.93	2.38	1.75	2.17	763.5	**0.010**
Combined foot dominance scale total mean left-footedness	6.30	6.65	5.03	6.75	619.5	0.279	7.33	6.90	4.15	6.29	715.5	**0.004**

^*^Mann–Whitney test. Significance at the 0.05 level (2-tailed).

#### Intra-gender comparisons

##### Male patients with schizophrenia vs. control males

The mean left-footedness is numerically higher in male patients with schizophrenia than the same-gender control subjects in 11 out of the 14 foot tests—Kick as high as possible, Kick a ball, Stamp out a cigarette, Balancing a rod on the thigh, Write your initials on the sand, Smooth the sand, Hopping on one foot, Tapping out a rhythm from the *Chapman & Chapman Subscale* and Step up on a chair, Pick up object with toe, and Standing on one foot from the *Complex Task Subscale* (Table 1).

Importantly, however, none of these differences is statistically significant at *p* < 0.05.

Neither the subtotal mean left-footedness of *Chapman & Chapman Subscale* (*p* = 0.288), nor the subtotal mean left-footedness of the *Complex Task Subscale* (*p* = 0.817) is statistically significantly higher in male patients with schizophrenia than the same-gender control subjects.

It should be emphasized that the total mean left-footedness of the *New Combined Foot Dominance Scale* is not statistically significantly higher in male patients with schizophrenia than in the same-gender control subjects (*p* = 0.279).

##### Female patients with schizophrenia vs. control females

In contrast to male subjects, there is a difference in female subjects.

- The mean left-footedness is numerically higher in female patients with schizophrenia than the same-gender controls in 12 out of the 14 foot tests—Kick as high as possible, Kick a ball, Rolling a golf ball around a circle, Stamp out a cigarette, Balancing a rod on the thigh, Smooth the sand, Hopping on one foot, Tapping out a rhythm from the *Chapman & Chapman Subscale* and Step up on a chair, Pick up object with toe, Push shovel into the ground, and Standing on one foot from the *Complex Task Subscale* (Table 1).
- Importantly, four of these differences are statistically significant at *p* < 0.05—Rolling a golf ball around a circle (*p* = 0.030), Balancing a rod on the thigh (*p* = 0.011) and Hopping on one foot (*p* = 0.011) from the *Chapman & Chapman Subscale* and Step up on a chair (*p* = 0.011) from the *Complex Task Subscale.*
- In contrast to males, a very slight opposite trend of higher mean left-footedness in controls is displayed in merely only one foot test—Write your initials on the sand from the *Chapman & Chapman Subscale* (*p* = 0.920, almost equal to the neutral *p* = 1.00). Arrange cubes from the *Chapman & Chapman Subscale* has equal means (0.38) in male patients with schizophrenia and same-gender controls (*p* = 0.991).
- Opposite to males, where neither subtotal mean exhibits statistically significant differences, a distinct pattern emerges in females. In female subjects, both subtotal mean left-footedness values reveal statistically significant differences. Specifically, the subtotal mean left-footedness of the Chapman & Chapman Subscale demonstrates a significantly higher value (4.40 vs. 2.4, *p* = 0.003, representing an increase of over 1.8 times). Additionally, the subtotal mean left-footedness of the Complex Task Subscale also shows significant differences (2.93 vs. 1.75, *p* = 0.010, representing an increase of over 1.6 times) at a significance level of *p* < 0.05.
- Most importantly, the total mean left-footedness of the *New Combined Foot Dominance Scale* in female patients with schizophrenia is strongly statistically significantly higher than in same-gender controls (7.33 vs. 4.15, *p* = 0.004, over 1.7 times increase).

### Comparison of mean left-footedness between male and female patients with schizophrenia and control group

Table 2 shows the male–female difference of mean left-footedness in 14 foot tests in both patients with schizophrenia and controls—in eight out of these 14 individual foot test, the differences are statistically stronger in the group of controls compared to the group of patients with schizophrenia. The item “Push shovel into the ground” reaches statistical significance—*p* < 0.004 in the control group.

**Table 2:** Comparison of *Mean Left-footedness* between male and female patients with schizophrenia and control group.

	Schizophrenia (*n* = 98)						Controls (*n* = 82)					
Foot tests	Men (*N* = 56)		Women (*N* = 42)		Statistical significance^*^		Men (*N* = 30)		Women (*N* = 52)		Statistical significance^*^	
	Mean	SD	Mean	SD	U	*p*	Mean	SD	Mean	SD	U	*p*
Chapman & Chapman subscale												
Kick as high as possible	0.41	0.78	0.52	0.89	1124.5	0.621	0.40	0.81	0.25	0.65	726.0	0.412
Kick a ball	0.30	0.71	0.33	0.75	1165.5	0.906	0.27	0.64	0.21	0.60	743.5	0.553
Arrange cubes	0.31	0.67	0.38	0.76	1110.5	0.806	0.43	0.77	0.38	0.77	745.5	0.652
Rolling a golf ball around circle	0.27	0.68	0.47	0.86	1043.0	0.226	0.27	0.69	0.15	0.54	736.0	0.410
Stamp out a cigarette	0.33	0.75	0.19	0.59	1053.0	0.313	0.27	0.69	0.12	0.47	721.0	0.241
Balancing a rod on the thigh	0.44	0.83	0.69	0.95	1000.5	0.148	0.31	0.71	0.25	0.65	726.5	0.660
Write your initials on the sand	0.25	0.67	0.14	0.52	1090.5	0.373	0.13	0.51	0.15	0.54	772.0	0.864
Smooth the sand	0.44	0.83	0.33	0.75	1095.5	0.529	0.23	0.63	0.25	0.65	777.5	0.968
Hopping on one foot	0.75	0.97	0.85	1.00	1092.0	0.590	0.50	0.78	0.37	0.77	690.0	0.248
Tapping out a rhythm	0.43	0.82	0.48	0.86	1111.0	0.816	0.20	0.55	0.27	0.69	772.0	0.896
Subtotal mean left-footedness	3.85	5.11	4.40	5.09	991.0	0.431	2.69	4.87	2.40	4.60	672.0	384
Complex Task Subscale												
Step up on a chair	0.87	1.00	0.86	1.00	1146.0	0.939	0.53	0.90	0.37	0.77	718.0	0.406
Pick up object with toe	0.42	0.80	0.43	0.83	1108.5	0.963	0.33	0.71	0.23	0.61	730.0	0.448
Push shovel into the ground	0.68	0.96	0.71	0.97	1155.0	0.855	1.03	0.10	0.42	0.82	534.5	0.004
Standing on one foot	0.57	0.79	0.93	0.95	912.5	0.068	0.47	0.73	0.73	0.91	674.0	0.240
Subtotal mean left-footedness complex tasks	2.40	2.05	2.93	0.2.38	964.5	0.322	2.37	2.19	1.75	2.17	627.0	0.126
Combined Foot Dominance Scale Total Mean Left-Footedness	6.30	6.65	7.33	6.90	958.5	0.469	5.03	6.75	4.15	6.29	628.0	0.206

^*^Mann–Whitney test.

Tables 3, 4 show the ANOVA models of the differences between four groups—Female-Controls, Male-Controls, Male-Schizophrenia, and Female-Schizophrenia in the three combined total scores of mean left-footedness, which we analyze in our study.

**Table 3:** ANOVA models of the differences between four groups—Females-Controls, Males-Controls, Males-Schizophrenia, and Females-Schizophrenia in three combined total scores of mean left-footedness.

		Sum of squares	df	Mean square	*F*	*p*
Subtotal mean left-footedness of 10 foot tests	Between groups	118,706	3	39,569	1.635	**0.183**
	Within groups	4,139,614	171	24,208		
	Total	4,258,320	174			
Subtotal mean left-footedness of four foot tests for complex task	Between groups	32,865	3	10,955	2.281	**0.081**
	Within groups	826,022	172	4,802		
	Total	858,886	175			
Total mean left-footedness of combined foot dominance scale of 14 foot tests	Between groups	267,195	3	89,065	2.031	**0.111**
	Within groups	7,411,568	169	43,855		
	Total	7,678,763	172			

Significance at the 0.05 level (2-tailed).

**Table 4:** Tukey HSD^a,b^ of homogeneous subsets.

Four group by gender-diagnosis	*N*	Subset for alpha = 0.05
		1
Females-controls	52	4.1538
Males-controls	29	5.0345
Males-schizophrenia	50	6.3000
Females-schizophrenia	42	7.3333
Sig.		0.135

Means for groups in homogeneous subsets are displayed.

^a^Uses harmonic mean sample size = 41,016.

^b^The group sizes are unequal. The harmonic mean of the group sizes is used. Type I error levels are not guaranteed.

In ANOVA analysis due to the statistical rules that the more groups compared, the greater the differences between them must be in order to reach statistical significance (*p* < 0.05) between them and avoid Type 1 error, i.e., finding difference between groups when there is not. The need for ANOVA arises from the error of alpha level inflation, which increases Type 1 error probability (false positive) and is caused by multiple comparisons.

We think that although none of our three combined total scores of mean left-footedness reach statistical significance of *p* < 0.05, this is due mainly to our relatively small sample size. The most significant difference is in subtotal mean left-footedness of four foot tests for Complex Task (*F*-2,28; *p* < 0.081), which approaches *p* < 0.05.

Table 4 shows that the difference between Females-Controls (4, 1,538) and Females-Schizophrenia (7, 3,333) is much higher than between Males-Controls (5, 0345) and Males-Schizophrenia (6, 3,000).

The most impressive difference is very logically for the ANOVA model showing that the differences between the four groups—Females-Controls, Males-Controls, Males-Schizophrenia, and Females-Schizophrenia in the Subtotal mean left-footedness of four foot tests for Complex Task *p* = 0.070, the strongest difference of the three means.

If we do not use the general alternative hypothesis, positing that the interaction Gender*Group may be possible in both way, i.e., stronger in men or stronger in women, and thus use two-tailed testing and postulate the specific alternative hypothesis that the interaction Gender*Group is logically higher in women than in men due to the fact that in the literature (24), female patients with schizophrenia are more left-footed than male patients with schizophrenia, then we can interpret *p* < 0.094 in the context of one-tailed testing as statistically significant interaction at *p* < 0.1. It is noteworthy to stress that studying the role of two factors in the variation of a certain variable, interpreting their interaction is obligatory and indispensable. In our case, the weakest statistical significance for the role of gender *per se* is even for the “Subtotal mean left-footedness of four foot tests for Complex Task” in comparison with the other two (Table 5). The lack of statistical significance for gender has been masked by the stronger role of women and the weaker role of men, neutralizing each other.

**Table 5:** UNIANOVA with interaction of subtotal mean left-footedness of 10 foot tests, subtotal mean left-footedness of four foot tests for complex task, and total mean left-footedness of combined foot dominance scale of 14 foot tests.

	Type III sum of squares	df	Mean square	*F*	*p*
Dependent variable: subtotal mean left-footedness of 10 foot tests					
GenderMen–Women	769	1	769	0.032	0.859
GroupControl–Schizophrenia	103,037	1	103,037	4.256	0.041
InteractionGender*Group	7,370	1	7,370	0.304	0.582
Dependent variable: subtotal mean left-footedness of four foot tests for complex task					
GenderMen-Women	88	1	88	0.018	0.892
GroupControl—Schizophrenia	15,460	1	15,460	3.219	0.075
InteractionGender*Group	13,627	1	13,627	2.837	**0.094**
Dependent variable: total mean left-footedness of combined foot dominance scale of 14 foot tests					
GenderMen-Women	1,215	1	1,215	0.039	0.843
GroupControl–Schizophrenia	99,236	1	99,236	3.221	0.078
InteractionGender*Group	12,083	1	12,083	0.392	0.534

Significance at the 0.05 level (2-tailed).

## Discussion

Our results show that patients with schizophrenia have markedly higher mean left-footedness than normal controls when not considering gender. However, the gender comparison reveals significant difference between females and males across different areas shown in the result section. The greatest proof of the female vs. male contribution to the overall higher difference in patients with schizophrenia vs. control subjects is given by comparing the subtotals mean left-footednesses and the total mean left-footedness by gender. In sharp contrast to females, neither the subtotals mean left-footednesses, nor the total mean left-footedness of the New Combined Foot Dominance Scale are statistically significantly higher in male schizophrenia patients vs. same-gender controls. Taken together, when considering the cumulative effect of all the aforementioned differences observed in the areas of comparison, shown in the results, it leads to the conclusion that only the females make a significant contribution to the overall higher mean left-footedness difference between patients with schizophrenia and control subjects. Thus, in terms of laterality schizophrenic dysontogenesis makes female patients with schizophrenia more lateralized than normally, matching normal males in that regard. Our findings of intra-gender difference in the mean left-footedness between female schizophrenia patients and control subjects in almost all tests correlate with the findings from magnetoencephalography-based studies also suggest anomalous cerebral lateralization in schizophrenia. In their study, Martin Reite et al. (24) found that the male patient subgroup exhibited significantly less asymmetry than the control group, while the female patient subgroup showed significantly more asymmetry. Further, in extending studies to female patients, data suggest that the nature of this anomaly is gender-specific.

Footedness can be identified with a combination of manipulation and stability tasks. The use of familiar tasks is more likely to elicit a stronger, more lateralized response. There is coherence between preference and performance across the stability modes, but preference for manipulation is substantially lateralized relative to performance (25). This could explain our findings in the four tests on the Complex task subscale showing a higher frequency of left foot manipulation-preference in female patients with schizophrenia. As well, the magnitude of the differences in performance abilities between the feet appears to depend on the task being examined. One of the reasons often cited is the degree of skill: the more complex the task the stronger the preference and the greater the preferred-foot advantage.

Because of abnormal brain asymmetry and failure of the left-hemisphere dominance in schizophrenia, lateralized functions are compromised in schizophrenia. Indeed, different studies have revealed abnormal patterns of connectivity in the left hemisphere in relation to specific psychotic domains (26–29). Left or mixed-handedness, footedness, and eye dominance are thought to indicate abnormalities in lateralization related to schizophrenia. Increased left or mixed dominance in schizophrenia suggests possible hemispheric abnormalities associated with the disorder (19). The New Combined Foot Dominance Scale allows for a subtler and comprehensive assessment of foot dominance in capturing the phenomenon of left-footedness. Besides, the total of 14 tests is administered as performance assessments, not as preference questionnaires.

In conclusion, the lateralized function in schizophrenia are compromised as a result of abnormal brain asymmetry and failure of the left-hemisphere dominance. Schizophrenia patients have markedly higher mean left-footedness than normal controls with a clear gender-specific effect, only notably pronounced in women not in men. Left foot dominance emerges as a promising indicator of altered hemispheric lateralization, as it is minimally influenced by cultural and traditional factors and reveals notable gender-specific differences.

## Limitations

The study is clinic-based and cross-sectional and not community-based, so there is certain selectivity bias in some Schizophrenia characteristics, as some representative demographics, severity of Schizophrenia process, but all such studies we encountered in literature are clinic-based and cross-sectional, because of exclusive difficulties of organizing such study as community-based and especially longitudinal.

## Data availability statement

The raw data supporting the conclusions of this article will be made available by the authors, without undue reservation.

## Ethics statement

The studies involving humans were approved by Medical University of Sofia, Department of Psychiatry and Medical Psychology. The studies were conducted in accordance with the local legislation and institutional requirements. The participants provided their written informed consent to participate in this study.

## Author contributions

KA: Conceptualization, Data curation, Formal analysis, Investigation, Methodology, Writing – original draft, Writing – review & editing.
